# Systolic and diastolic function in chronic spinal cord injury

**DOI:** 10.1371/journal.pone.0236490

**Published:** 2020-07-27

**Authors:** Bonnie Legg Ditterline, Shelley Wade, Beatrice Ugiliweneza, Narayana Sarma V. Singam, Susan J. Harkema, Marcus F. Stoddard, Glenn A. Hirsch

**Affiliations:** 1 Kentucky Spinal Cord Injury Research Center, University of Louisville, Louisville, KY, United States of America; 2 Department of Neurological Surgery, University of Louisville, Louisville, KY, United States of America; 3 Department of Health Management and Systems Science, University of Louisville, Louisville, KY, United States of America; 4 Division of Cardiovascular Medicine, Department of Medicine, University of Louisville, Louisville, KY, United States of America; University of Rome, ITALY

## Abstract

Individuals with spinal cord injury develop cardiovascular disease more than age-matched, non-injured cohorts. However, progression of systolic and diastolic dysfunction into cardiovascular disease after spinal cord injury is not well described. We sought to investigate the relationship between systolic and diastolic function in chronic spinal cord injury to describe how biological sex, level, severity, and duration of injury correlate with structural changes in the left ventricle. Individuals with chronic spinal cord injury participated in this study (n = 70). Registered diagnostic cardiac sonographers used cardiac ultrasound to measure dimensions, mass, and systolic and diastolic function of the left ventricle. We found no significant relationship to severity or duration of injury with left ventricle measurements, systolic function outcome, or diastolic function outcome. Moreover, nearly all outcomes measured were within the American Society of Echocardiography-defined healthy range. Similar to non-injured individuals, when indexed by body surface area (BSA) left ventricle mass [-14 (5) g/m2, p < .01], end diastolic volume [-6 (3) mL/m^2^, p < .05], and end systolic volume [-4 (1) mL/m^2^, p < .01] were significantly decreased in women compared with men. Likewise, diastolic function outcomes significantly worsened with age: E-wave velocity [-5 (2), p < .01], E/A ratio [-0.23 (0.08), p < .01], and e’ velocity [lateral: -1.5 (0.3) cm/s, p < .001; septal: -0.9 (0.2), p < .001] decreased with age while A-wave velocity [5 (1) cm/s, p < .001] and isovolumic relaxation time [6 (3) ms, p < .05] increased with age. Women demonstrated significantly decreased cardiac size and volumes compared with men, but there was no biological relationship to dysfunction. Moreover, individuals were within the range of ASE-defined healthy values with no evidence of systolic or diastolic function and no meaningful relationship to level, severity, or duration of injury. Decreases to left ventricular dimensions and mass seen in spinal cord injury may result from adaptation rather than maladaptive myocardial remodeling, and increased incidence of cardiovascular disease may be related to modifiable risk factors.

## Introduction

Cardiac myocyte atrophy and decreased left ventricular mass and dimensions are well-documented in spinal cord injury [1–5] and likely result in decreased ejection fraction and cardiac output [6–9]. Chronic skeletal muscle unloading can rapidly decrease left ventricular volumes, mass, and contraction velocity [10–16], while sympathetic impairment decreases myocardial contractility and cardiac response to stress or exercise [17–22]. Decreased systolic and diastolic functional outcomes (e.g., ejection fraction and passive diastolic filling) in the non-injured population illustrate maladaptive cardiac remodeling and thus carry significant risk of cardiovascular disease [23–25], and individuals with spinal cord injury develop cardiovascular disease younger and more frequently than age-matched, non-injured cohorts [26–31]. Despite this, effects of spinal cord injury on manifest systolic and diastolic dysfunction remains unclear. Decreased ejection fraction and cardiac output aside, there is little data available on clinical measures of systolic function (e.g., strain imaging, dP/dt) in human spinal cord injury, though animal models illustrate impairment [32, 33]. Other research groups have reported diastolic dysfunction in quadriplegia and paraplegia compared to able-bodied controls and athletes with spinal cord injury [1, 2, 34, 35]; others have found therapeutic interventions can increase chamber size and improve diastolic filling ratios [36–38], suggesting a spinal cord injury-related deficit. Confounding this, others have found decreased left ventricular dimensions and mass do not correlate with systolic or diastolic dysfunction in spinal cord injury [3, 4, 39].

It is thus unclear how decreased left ventricular outcomes relate to systolic and diastolic dysfunction in spinal cord injury: is the decreased ejection fraction and decreased passive filling a clinical marker that predicts early onset of systolic and diastolic dysfunction in spinal cord injury, or is this a functional adaptation to hypotension, hypovolemia, and decreased metabolic demand? There are no large, representative sample cohort studies that attempt to elucidate the effects of spinal cord injury on cardiac function. Moreover, women are drastically underrepresented in biomedical research, and spinal cord injury is no different [40–42]: previous research reported herein include outcomes obtained from 371 individuals with spinal cord injury, of whom seven are female. In 2001, the National Committee for Medicine reported differences in cardiovascular disease manifest symptoms, risk factors, and mortality between women and men can be attributed to biological sex [43]. Investigation into cardiac function in women with spinal cord injury is thus an important contribution to the field in order to understand sex-related changes and predict cardiovascular disease risk. We therefore sought to investigate how biological sex and spinal cord injury affect cardiac function. We hypothesized, first: women with spinal cord injury would have smaller cardiac dimensions and volumes, but better systolic and diastolic function outcomes, similar to the non-injured population; second, left ventricle chamber size, systolic function, and diastolic function would worsen with prolonged immobility, and as such outcomes would significantly worsen as duration of injury increased.

## Materials and methods

### Participant recruitment

This descriptive study enrolled 70 (Table 1) North American individuals with traumatic spinal cord injury. Inclusion criteria were as follows: adults with chronic spinal cord injury; medically stable, with no ongoing or history of manifest cardiovascular disease, diabetes mellitus, chronic hypertension, hyperlipidemia, or thrombi; no history of drug abuse; and no ongoing tobacco use. All individuals reported cardiovascular impairment related to spinal cord injury, including, but not limited to, persistent hypotension, orthostatic hypotension, and/or episodes of autonomic dysreflexia. Mean age of individuals enrolled was 36 ± 12 years (Table 2) and mean duration of injury was 8 ± 7 years; 51 individuals were male. Females had a significantly decreased body surface area (BSA) compared with males (p < .001); no other demographic differences were found. To test effects of spinal cord injury on cardiac outcomes, individuals were divided into cervical and thoracic spinal cord injury. This research study was approved by the University of Louisville Institutional Review Board in accordance with the Declaration of Helsinki. Individuals provided written, informed consent in order to participate (NCT-02037620 and NCT-03364660).

**Table 1:** Demographics of individuals.

Individual	Age (years)	Sex	Duration of Injury (years)	Level of Injury	AIS Grade	BSA (m^2^)	SBP (mmHg)	DBP (mmHg)	HR (BPM)
A68	34.6	M	3.5	C1	A	1.75	87	48	45
A80	32.6	F	7.6	C1	A	1.67	.	.	60
B53	32.5	M	14.2	C1	B	2.02	91	55	43
A102	26.9	F	2.2	C2	A	1.76	96	51	74
A88	55.6	M	11.1	C2	A	2.13	100	51	65
B46	71.4	F	6.3	C2	B	2.01	145	85	38
C33	48.5	M	44.4	C2	C	1.81	.	.	.
C55	49.2	M	5.0	C2	C	2.41	.	.	.
D38	42.3	M	13.0	C2	D	2.26	.	.	.
A101	30.7	M	1.7	C3	A	2.00	109	54	44
A113	27.2	M	6.4	C3	A	1.92	84	38	53
A121	51.2	M	3.4	C3	A	2.09	90	54	69
A91	23.8	F	4.8	C3	A	1.62	81	48	57
A100	50.7	M	15.4	C4	A	2.15	87	50	68
A104	43.5	F	7.1	C4	A	1.72	105	60	45
A105	33.0	M	9.3	C4	A	1.96	128	62	47
A109	40.5	M	14.2	C4	A	1.67	97	59	69
A114	24.2	M	13.0	C4	A	1.79	117	74	60
A116	25.6	F	9.3	C4	A	1.41	96	61	65
A58	40.2	M	1.9	C4	A	2.22	.	.	59.9
A64	53.3	M	36.2	C4	A	2.04	123	92	49
A65	32.2	M	1.5	C4	A	1.95	.	.	.
A67	30.1	F	3.8	C4	A	1.68	.	.	.
A81	45.9	M	2.7	C4	A	2.40	138	65	47
A86	31.9	M	10.9	C4	A	1.82	105	61	43
A96	25.9	F	2.0	C4	A	1.76	130	63	40
A97	35.1	M	11.8	C4	A	1.77	85	42	43
A99	19.0	M	1.9	C4	A	1.79	80	47	43
C54	28.2	M	6.7	C4	A	2.17	113	69	60
A120	33.8	M	13.4	C4	B	1.75	75	47	50
B29	40.5	F	9.2	C4	B	1.79	82	48	80
B32	59.8	M	6.6	C4	B	1.82	86	51	.
B38	19.9	M	1.2	C4	B	2.33	.	.	50
B42	55.3	M	4.0	C4	B	2.07	117	68	56
B43	30.7	M	1.9	C4	B	2.02	107	62	53
B47	42.2	M	7.1	C4	B	2.01	95	51	34
B48	27.9	F	8.1	C4	B	1.80	77	40	59
B49	28.8	M	3.3	C4	B	2.22	.	.	.
B51	25.4	M	3.5	C4	B	1.84	129	70	75
C27	58.1	M	5.4	C4	C	2.04	.	.	.
C40	34.4	M	9.0	C4	C	2.01	.	.	.
A106	23.3	M	5.3	C5	A	1.94	102	50	49
A108	31.3	F	4.0	C5	A	1.60	88	53	50
A119	23.9	F	8.9	C5	A	1.42	89	57	57
A41	23.9	M	7.1	C5	A	2.07	100	59	48
B45	33.0	M	7.0	C5	B	2.06	161	76	45
A118	21.7	F	7.7	C6	A	1.56	126	79	67
B24	24.5	M	5.7	C6	B	1.54	92	45	53
B44	41.3	M	24.8	C6	B	2.09	117	69	40
B52	49.9	F	6.4	C6	B	1.58	102	58	60
A110	21.3	F	5.2	C7	A	2.18	97	54	54
B28	39.1	M	9.5	C7	A	1.86	105	53	56
B31	25.4	M	9.3	C7	B	1.77	120	76	48
B50	32.5	M	4.0	C7	B	1.95	119	73	67
B54	25.5	F	3.6	C7	B	1.67	98	55	64
A112	22.7	M	4.0	C8	A	2.22	103	52	40
B41	25.7	M	7.5	C8	B	2.17	117	62	86
A103	37.1	M	1.9	T1	A	1.77	91	53	56
A111	30.5	M	5.2	T2	A	1.60	99	55	56
A117	27.9	M	5.9	T2	A	1.95	92	51	63
C37	19.8	F	6.7	T2	C	1.75	94	50	69
A107	67.6	F	9.8	T3	A	1.59	155	67	65
A46	50.8	F	9.5	T3	A	2.00	94	51	61
B34	25.6	M	1.9	T4	B	1.99	114	63	53
C44	39.9	M	1.6	T4	B	2.05	123	78	78
A122	46.9	M	10.3	T5	C	2.42	102	60	54
A115	50.9	M	14.5	T6	A	1.66	112	71	69
A61	48.5	M	2.0	T6	A	2.01	92	54	43
A94	35.1	M	4.7	T7	A	2.18	102	59	58
A55	38.9	M	8.1	T10	A	1.98	118	73	86

Demographics are reported with respect to when echocardiographic measurements were obtained. AIS: ASIA Impairment Scale; C: cervical; T: thoracic; BSA: body surface area; SBP: systolic blood pressure; DBP: diastolic blood pressure; HR: heart rate; BPM: beats per minute. Blank SBP and DBP values were not recorded during assessment.

**Table 2:** Demographic summary of individuals.

		All Participants	Female	Male	p-value
		(n = 70)	(n = 19)	(n = 51)	
**Age (years)**					
	Median (Q1, Q3)	33 (26, 44)	28 (24, 44)	35 (28, 46)	0.191
**Sex**					
	Female, n (%)	19 (27.14%)			
	Male, n (%),	51 (72.86%)			
**Duration of Injury (years)**					
	Median (Q1, Q3)	6.5 (3.6, 9.4)	6.7 (4, 8.9)	6.4 (3.4, 10.9)	0.916
**BSA (m**^**2**^**)**					
	Mean (SD)	1.9 (0.2)	1.7 (0.2)	2 (0.2)	< .0001
**Level of Injury**					0.591
	Cervical, n (%)	56 (80%)	16 (84%)	40 (78%)	
	Thoracic, n (%)	14 (20%)	3 (16%)	11 (22%)	
**Completeness of injury**					0.307
	AIS Grade A, n (%)	41 59%)	13 (68%)	28 (55%)	
	AIS Grade B, C, or D n (%)	29 (41%)	6 (32%)	23 (45%)	
**SBP (mmHg)**					
	Median (Q1, Q3)	102 (91, 117)	96 (89, 105)	103 (92, 117)	0.439
	Missing	11	2	9	

Demographics are reported with respect to when echocardiographic measurements were obtained. BSA: body surface area; AIS: ASIA Impairment Scale; SBP: systolic blood pressure.

### Echocardiography

Acquisition and analysis: Individuals were passively moved to the left-lateral decubitus position and given sufficient time to acclimate prior to recording. Brachial blood pressure was recorded from the right arm. Individuals did not consume caffeine, alcohol, or blood pressure medication the morning of the exam; abdominal binders and compression garments were removed. Registered diagnostic cardiac sonographers recorded images on a Philips EPIQ 7 ultrasound system with a Philips X5-1 MHz xMATRIX array transducer or a GE LOGIQ P6 ultrasound system with a GE 3Sp-D phased array transducer. Images were obtained in the parasternal long axis, parasternal short axis, and apical 2-, 3-, 4- and 5-chamber views according to the standards and recommendations of the American Society of Echocardiography (ASE) [44, 45]. Aortic, left ventricular, and left atrial dimensions were obtained using 2-dimensional echocardiography; left ventricular outflow velocities were measured using pulsed-wave Doppler recorded from the left ventricular outflow tract and aortic annulus; mitral inflow velocities during early (E-wave) and late (A-wave) diastole were recorded from mitral valve leaflet tips using pulsed-wave Doppler; isovolumic relaxation time was measured as the time between aortic valve closure and mitral valve opening using pulsed-wave Doppler of aortic outflow and mitral inflow; myocardial peak early diastolic relaxation velocity was measured using tissue Doppler imaging in the lateral and septal annulus during early (e′ velocity) diastole. Four consecutive cardiac cycles were recorded for off-line analysis.

Images were analyzed according to the standards and recommendations of the ASE; likewise, dimensions and volumes, where appropriate, are indexed to body surface area (BSA) [44, 45]. Left ventricular volumes were estimated using Simpson’s biplane method of discs from images obtained in the apical 2- and 4-chamber views in end-systole and end-diastole. Cardiac output and stroke volume were calculated from left ventricular outflow tract diameter and velocity time integral (VTI) of blood flow measured from the parasternal long axis and 5-chamber views, respectively. Left ventricular mass was calculated from internal diastolic diameter, posterior wall dimension, and septal dimension. Relative wall thickness of the left ventricle is calculated as the ratio of twice the posterior wall dimension to internal diastolic diameter. Global longitudinal strain was calculated from apical 2-, 3-, and 4-chamber views. Global circumferential strain was measured from the parasternal short-axis view at basal-, mid- and apical-depths. Cardiac measurements were compared to the healthy guidelines established by the ASE.

### Statistics

Body surface area was normally distributed and was summarized with mean and standard deviation. Age, duration of injury and systolic blood pressure were skewed and were summarized with median and interquartile range. Biological sex and level of injury (cervical and thoracic) were summarized with frequency count and percentages. Systolic blood pressure (mmHg) was included in the model as an index of sympathetic regulation (reflected by increasing systolic blood pressure) [21]. We analyzed the effect of demographic data, systolic blood pressure, duration of injury, and severity of injury on screening cardiac measurements and functional outcomes using linear models. All those factors were used as fixed effects. Systolic blood pressure (mmHg) was included as a covariate to illustrate effects of increasing spinal sympathetic outflow (reflected by increasing systolic blood pressure) on cardiac structure and function. Cardiac measurements were indexed by BSA and as such BSA was not included as a covariate in the model. We were interested in effects of biological sex (male vs female), level of injury (cervical vs thoracic), duration of injury (years), and severity of injury (increasing systolic blood pressure) on cardiac outcomes. To obtain them, we built linear contrasts in the modeling process. Estimates of the difference attributed to each covariate was presented as least squares means estimates with their associated 95% confidence interval. Z-scores were calculated for each observation ($\frac{value-mean_{normative}}{standard\;deviation_{normative}}$) to compare the skew of our data to normative echocardiography data obtained from ASE datasets. VTI measured at the left ventricular outflow tract does not have a healthy range established by the ASE–only a minimum healthy value–and as such is the only outcome without an associated Z-score. All tests were 2-sided with a significance level of 0.05. Model results are reported as beta-coefficient (SE) when p < .05. SAS 9.4 (SAS Institute, Cary, NC) was used for data analysis.

## Results

### Chamber dimensions, geometry, and mass

**Fig 1:**
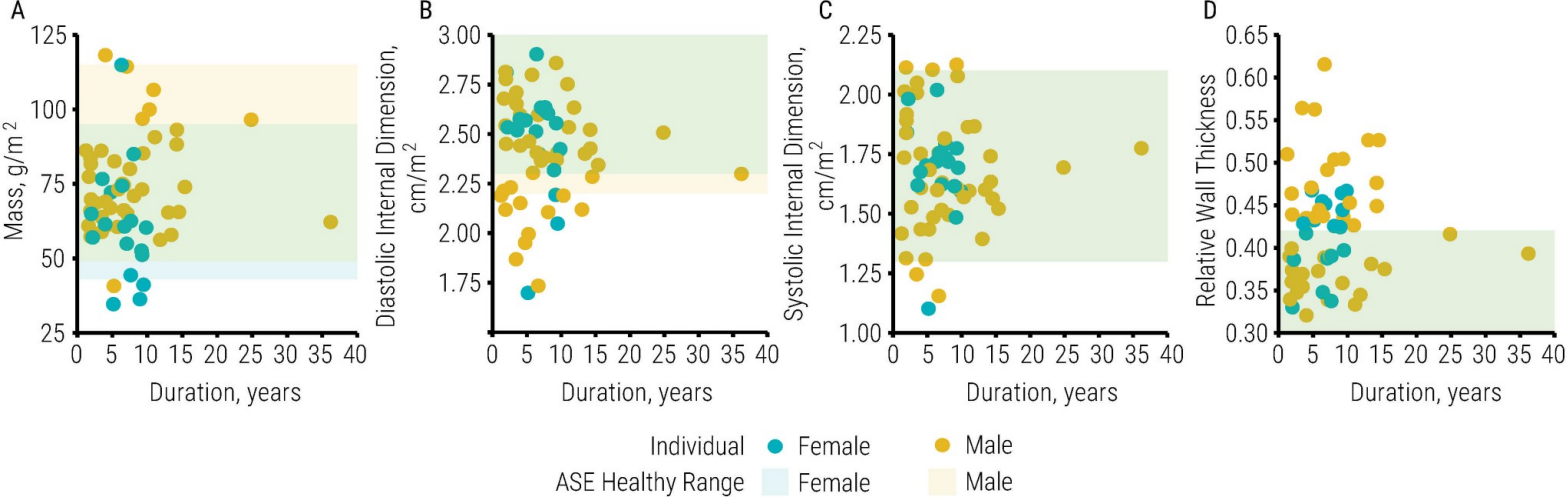
Left ventricular measurements in individuals with spinal cord injury. Illustrated is left ventricular mass indexed by body surface area (BSA; A), diastolic internal dimension indexed by BSA (B), systolic internal dimension indexed by BSA (C), and relative wall thickness (D) in individuals with spinal cord injury (n = 70) as duration of injury increases. Female values are blue, male values are yellow; circles illustrate individual datapoints, while rectangles illustrate the range of healthy values as determined by the American Society of Echocardiography. Only left ventricular mass was significantly less in women compared with men [-14 (4) g/m^2^, p < .01]. There was no significant relationship between left ventricular measurements and duration of injury, and most left ventricular measurements fell within the range of healthy values. Data are reported as beta-coeffecient (SE); significance was set to p < .05.

Cardiac dimensions and structure in women were significantly less compared with men (Table 3), but there were no significant relationships between cardiac dimensions and duration of injury or systolic blood pressure. Moreover, nearly all left ventricular measurements were within the healthy range as determined by the ASE (Fig 1, S1 Table in S1 data).

**Table 3:** Left side chamber size, geometry, and mass values and multivariate model results with ASE normal values.

		ASE Normal Limits		SCI Individuals (n = 70)							Beta-Coefficient (SE) for Multivariate Models				
		Female	Male	Female			Male			p-value, female vs male	Female vs male	Age (10-year increase)	Duration of injury (1-year increase)	Thoracic vs cervical level of injury	SBP (5 mmHg difference)
				Mean±SE	Min	Max	Mean±SE	Min	Max						
**Left Ventricular Dimensions**															
	Left Ventricle Internal Diameter during Diastole, cm	3.8–5.2	4.2–5.8	4.1±0.1	3.3	5.1	4.6±0.1	3.2	5.4	0.0005	-0.5 (0.1)			-0.4 (0.2)	
	Left Ventricle Internal Diameter during Diastole indexed to BSA, cm/m^2^	2.3–3.1	2.2–3.0	2.4±0.1	1.7	2.9	2.4±0.1	1.7	2.9					-0.2 (0.1)	
	Left Ventricle Internal Diameter during Systole, cm	2.8–3.3	2.5–4.0	2.8±0.1	2.3	3.5	3.3±0.1	2.3	4.3	0.0021	-0.4 (0.1)				
	Left Ventricle Internal Diameter during Systole indexed to BSA, cm/m^2^	1.3–2.1	1.3–2.1	1.7±0.1	1.1	2.0	1.7±0.1	1.2	2.1						
	Relative Wall Thickness, cm	0.22–0.42	0.24–0.42	0.44±0.02	0.33	0.47	0.44±0.01	0.32	0.62					0.1 (0.02)	
	Interventricular Septum Dimension, cm	0.60–0.90	0.60–1.00	0.76±0.05	0.45	1.2	0.86±0.03	0.52	1.5			0.05 (0.02)			
	Left Ventricle Posterior Wall Dimension during Diastole, cm	0.60–0.90	0.60–0.90	0.89±0.03	0.70	1.2	0.99±0.02	0.80	1.3	0.0041	-0.1 (0.03)				
**Aortic and Left Atrial Dimensions**															
	Aortic Root Diameter, cm	2.4–3.6	2.8–4.0	2.7±0.1	2.3	3.3	3.2±0.1	2.3	4.2	< .0001	-0.4 (0.1)	0.2 (0.04)			
	LVOT Diameter, cm	1.6–2.3	1.7±2.6	2.0±0.1	1.6	2.2	2.2±0.1	1.9	3.0	< .0001	-0.3 (0.1)				
	Left Atrial Dimension, cm	2.70–3.80	3.00–4.00	2.9±0.1	1.6	4.1	2.9±0.1	1.9	4.4			0.2 (0.06)		0.4 (0.2)	
	Left Atrial Dimension indexed to BSA, cm/m^2^	1.50–2.30	1.50–2.30	1.7±0.1	1.1	2.1	1.5±0.1	1.0	1.8	0.004	0.2 (0.1)	0.1 (0.03)		0.2 (0.1)	
**Left Ventricle Mass, End Diastole**															
	Left Ventricle Mass from Linear Method, g	67–162	88–224	101±10	52	231	145±6	65	256	0.0002	-44 (11)	14 (4)			
	Left Ventricle Mass from Linear Method indexed to BSA, g/m^2^	43–95	49–115	59±4	35	115	73±s3	41	118	0.0045	-14 (5)	6 (2)		-11 (5)	

ASE: American Society of Echocardiography; SCI: Spinal Cord Injury; BSA: body surface area; LVOT: left ventricular outflow tract.

In the left ventricle, internal dimension at end-diastole [-0.53 (0.14) cm, p < .001], internal dimension at end-systole [-0.42 (0.13) cm, p < .01], and posterior wall dimension [-0.10 (0.03), p < .01] were significantly smaller in women compared with men. Diameter of the aortic root [-0.43 (0.1) cm, p < .001] and left ventricular outflow tract [-0.26 (0.06) cm, p < .001] were also significantly smaller in women compared with men. Indexed by BSA, left ventricular internal diameter during diastole and systole were no longer significant, but left atrial dimension [0.20 (0.06) cm/m^2^, p < .01] in women was significantly greater compared with men. Left ventricular mass [absolute: -44 (11) g, p < .001; indexed by BSA: -14 (5) g/m^2^, p < .01] in women was significantly smaller compared with men.

Interventricular septum dimension increased significantly with age [0.05 (0.02) cm, p < .05 ], as did aortic root diameter [0.16 (.04) cm, p < .001], left atrial dimension [absolute: 0.16 (0.06) cm, p < .01; indexed by BSA: 0.06 (0.03) cm/m^2^, p < .05 ], and left ventricular mass [absolute: 14 (4) g, p < .01 ; indexed by BSA: 6 (2) g/m^2^, p < .05].

In thoracic injuries, left ventricle internal dimension at end-diastole [absolute: -0.4 (0.2) cm, p < .05; indexed by BSA: -0.2 (0.1) cm/m^2^, p < .05] and mass [indexed by BSA: -11 (5) cm/m^2^, p < .05] were significantly less compared with cervical injuries. Relative wall thickness [0.1 (0.02), p < .05] and left atrial dimension [absolute: 0.4 (0.2) cm, p < .05; indexed by BSA: 0.2 (0.1) cm/m^2^, p < .01] were significantly greater in thoracic injuries compared with cervical. In men, cardiac output indexed by BSA was below the healthy ASE range; in women, cardiac output, absolute and indexed by BSA, and absolute stroke volume was below the ASE healthy range.

Finally, the only left ventricular measurement in our spinal cord injury group above the healthy range determined by the ASE was posterior wall dimension and relative wall thickness; Z-scores indicate these values in male individuals with spinal cord injury are skewed when compared with the normative values. Left atrial dimension absolute and indexed by BSA was below the ASE healthy range, and Z-scores indicate these values are skewed in male individuals with spinal cord injury when compared with the normative values.

### Systolic function and blood pressure

**Fig 2:**
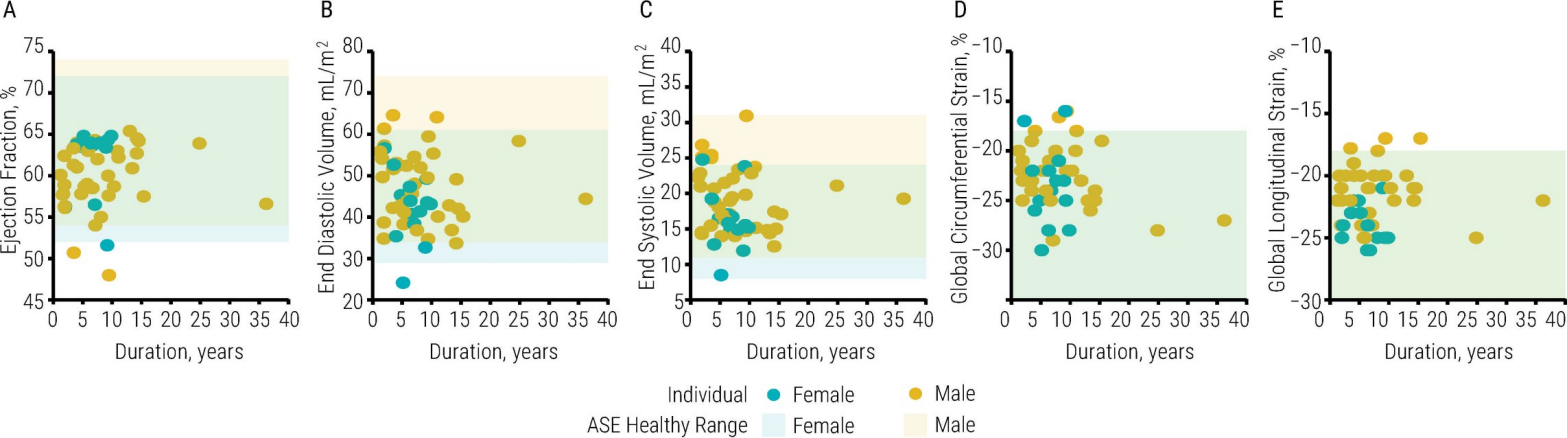
Global systolic function outcomes in individuals with spinal cord injury. Illustrated is ejection fraction (A), end diastolic volume indexed to body surface area (BSA; B), end systolic volume indexed to BSA (C), global circumferential strain (D), and global longitudinal strain (E) in individuals with spinal cord injury (n = 70) as duration of injury increases. Female values are blue, male values are yellow; circles illustrate individual datapoints, while rectangles illustrate the range of healthy values as determined by the American Society of Echocardiography. End diastolic volume [-6 (3) mL/m2, p < .05], end systolic volume [-4 (1) mL/m2, p < .01], and global longitudinal strain [-2 (0.7) %, p < .01] were significantly less in women compared with men. There was no significant relationship to duration of injury, and most left ventricular measurements fell within the range of healthy values. Data are reported as beta-coeffecient (SE); significance was set to p < .05.

Most global systolic function outcomes were significantly different between women and men (Table 4). There were no significant relationships between global systolic function outcomes and duration of injury or systolic blood pressure, and few significant relationships between systolic function outcomes and level of injury. Moreover, nearly all systolic function outcomes were within the healthy range as determined by the ASE (Fig 2, S2 Table in S1 data).

**Table 4:** Global systolic function and blood pressure outcomes and multivariate model results with ASE normal values.

		ASE Normal Limits		SCI Individuals (n = 70)							Beta-Coefficient (SE) for Multivariate Models				
		Female	Male	Female			Male			p-value, female vs male	Female vs male	Age (10-year increase)	Duration of injury (1-year increase)	Thoracic vs cervical level of injury	SBP (5 mmHg difference)
				Mean±SE	Min	Max	Mean±SE	Min	Max						
Left Ventricular Volumes, Simpson's Biplane Method															
	Ejection Fraction, %	54–74	52–72	62±1	52	65	60±1	48	65						
	End Diastolic Volume, mL	46–106	62–150	69±5	47	100	92±3	56	134	0.0003	-23 (6)				
	End Diastolic Volume indexed to BSA, mL/m^2^	29–61	34–74	40±3	24	57	47±2	34	65	0.0264	-6 (3)				
	End Systolic Volume, mL	14–42	21–61	26±3	17	44	37±2	21	57	0.0006	-11 (3)				
	End Systolic Volume indexed to BSA, mL/m^2^	8–24	11–31	15±1	9	25	19±1	13	31	0.0172	-3 (1)				
**Functional Outcomes**															
	Velocity Time Integral, cm	≥18	≥18	22±1	14	34	21±1	11	36					-3 (2)	
	Cardiac Output, L/min	4.0–8.0	4.0–8.0	4.0±0.3	2	6	4.5±0.2	2	9.6						
	Cardiac Output indexed to BSA, L/min/m^2^	2.4–4.2	2.4–4.2	2.3±0.2	1.1	3.6	2.3±0.1	1.1	4.7						
	Stroke Volume, mL	70–100	70–100	65±6	41	107	79±4	46	141	0.0418	-14 (7)	5 (3)			
	Stroke Volume, indexed to BSA, mL/m^2^	32–58	32–58	38±3	23	56	40±2	25	70						
	Systolic Blood Pressure, mmHg	<120	<120	104±5	77	155	104±3	75	138						NA
	Diastolic Blood Pressure, mmHg	<80	<80	59±3	40	85	59±2	38	92						NA
	Heart Rate, BPM	50–100	50–100	62±3	38	80	58±2	34	86					9 (4)	NA
**Strain**															
	Global Circumferential	≥-20	≥-20	-24±1	-30	-16	-22±1	-29	-16						
	Global Longitudinal	≥-20	≥-20	-24±1	-26	-20	-21±1	-25	-17	0.003	-2 (1)				

End diastolic volume [absolute: -23 (6) mL, p < .001; indexed by BSA: -6 (3) mL/m^2^, p < .05], end systolic volume [absolute: -11 (3) mL, p < .01; indexed by BSA: -3 (1) mL/m^2^, p < .01], stroke volume [-14 (7) mL, p < .05], and global longitudinal strain [-2 (1) %, p < .01] were significantly less in women compared with men.

In thoracic injuries, heart rate [9 (4) BPM, p < .05] was significantly greater and VTI [-3 (2), p < .001] was significantly less compared with cervical injuries. Stroke volume [5 (3) mL, p < .05] increased significantly with age. In men, cardiac output indexed by BSA was below the healthy ASE range; in women, cardiac output, absolute and indexed by BSA, and absolute stroke volume was below the ASE healthy range. The Z-scores associated with cardiac output indexed by BSA indicate these values are skewed in female and male individuals with spinal cord injury when compared with normative values.

### Diastolic function

**Fig 3:**
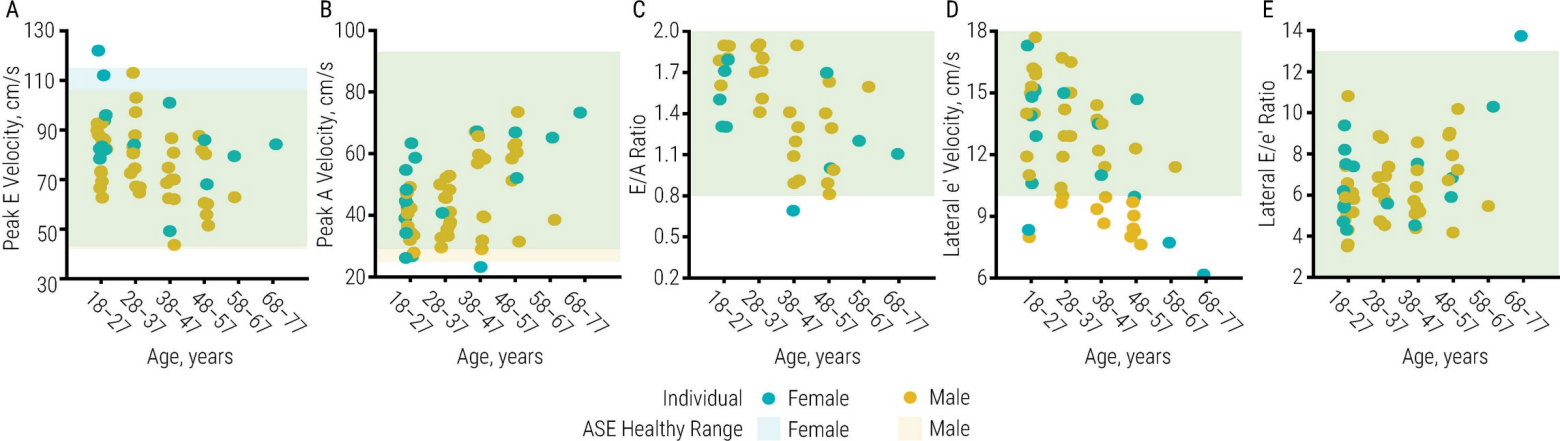
Global diastolic function and age in individuals with spinal cord injury. Illustrated is ejection fraction (A), end diastolic volume (B), end systolic volume (C), global circumferential strain (D), and global longitudinal strain (E) in individuals with spinal cord injury (n = 70) as duration of injury increases. Female values are blue, male values are yellow; circles illustrate individual datapoints, while rectangles illustrate the range of healthy values as determined by the American Society of Echocardiography. No outcomes were significantly less in women compared with men. Mitral valve A-wave velocity [5 (1) cm/s, p < .001] and lateral E/e’ ratio [lateral: 0.7 (0.2), p < .001] increased significantly with age. Mitral E-wave velocity [-5 (2), p < .01], E/A ratio [-0.2 (0.1), p < .01], and e’-wave velocity [lateral: -1.5 (0.3) cm/s, p < .001] decreased significantly as age increased. Data are reported as beta-coefficient (SE); significance was set to p < .05.

There were no significant relationships between diastolic function and duration of injury or biological sex. Most differences in diastolic function were significantly related to age (Table 5), and nearly all diastolic function outcomes were within the healthy range as determined by the ASE (Fig 3, S3 Table in S1 data).

**Table 5:** Diastolic function outcomes and multivariate model results with ASE normal values.

		ASE Normal Limits		SCI Individuals (n = 70)							Beta-Coefficient (SE) for Multivariate Models				
		Female	Male	Female			Male			p-value, female vs male	Female vs male	Age (10-year increase)	Duration of injury (1-year increase)	Thoracic vs cervical level of injury	SBP (5 mmHg difference)
				Mean±SE	Min	Max	Mean±SE	Min	Max						
**Mitral Valve Velocitiess**															
	Mitral Valve Peak E-wave Velocity, cm/s	43–115	42–106	83±4	49	122	75±3	44	133			-5 (2)		-10 (5)	
	Mitral Valve Peak A-wave Velocity, cm/s	29–93	25–93	51±3	23	73	46±2	28	74			5 (1)			
	E/A Wave Ratio	≥0.8	≥0.8	1.8±0.2	0.7	4.3	1.7±0.1	0.8	4.0			-0.2 (0.1)		-0.6 (0.2)	
	Mitral Valve Deceleration Time, ms	140–240	140–240	183±12	134	223	225±8	145	337	0.0018	-42 (13)				
	Isovolumic Relaxation Time, ms	70–100	70–100	87±6	64	146	94±4	60	156			6 (3)			
**Relaxation Velocity**															
	e' Velocity, cm/s, measured Laterally	≥10	≥10	12±1	6.2	19	13±1	7.6	19			-1.5 (0.3)			
	Lateral E/e' ratio	≤13	≤13	6.7±0.4	4.3	14	6.0±0.3	3.5	11			0.7 (0.2)		-1.1 (0.5)	0.2 (0.1)
	e' Velocity, cm/s, measured Septally	≥8	≥8	9.6±0.5	4.2	14	10±1	6.0	14			-0.9 (0.2)			
	Septal E/e' ratio	≤15	≤15	8.9±0.6	5.5	20	7.4±0.4	4.9	13	0.0349	1.5 (0.7)	0.6 (0.3)		-1.9 (0.7)	0.3 (0.1)

ASE: American Society of Echocardiography; SCI: Spinal Cord Injury.

Only mitral valve deceleration time [-42 (13) ms; p < .01] was significantly less in women compared with men. Septal E/e’ ratio [1.5 (0.7), p < .05] was significantly greater in women compared with men. Mitral valve A-wave velocity [5 (1) cm/s, p < .001], isovolumic relaxation time [6 (3) ms, p < .05], and E/e’ ratio [lateral: 0.7 (0.2), p < .001; septal: 0.6 (0.3), p < .05] increased significantly with age. Mitral E-wave velocity [-5 (2), p < .01], E/A ratio [-0.25 (0.08), p < .01], and e’-wave velocity [lateral: -1.5 (0.3) cm/s, p < .001; septal -0.88 (0.21) cm/s, p < .001] decreased significantly with age.

In thoracic injuries, mitral valve peak E-wave velocity [-10 (5) cm/s, p < .05], E/A ratio [-0.6 (0.2) cm/s, p < .05], and E/e’ ratio [lateral: -1.1 (0.5), p < .05; septal: -1.9 (0.7), p < .05] were significantly less compared with cervical injuries. E/e’ ratio [lateral: 0.2 (0.1), p < .05; septal: 0.3 (0.1), p < .01] increased significantly as systolic blood pressure increased. Mitral valve deceleration time in male individuals with spinal cord injury was greater than the ASE healthy values, and the associated Z-score indicates these values are skewed when compared with normative values.

## Discussion

We report individuals with chronic spinal cord injury do not experience systolic and diastolic dysfunction despite profound cardiovascular dysregulation. There were significant differences between men and women with spinal cord injury in cardiac structure and chamber volumes, but systolic and diastolic function outcomes were not significantly different in women compared with men. Diastolic function outcomes significantly decreased with age, similar to the non-injured population, indicating declines in function may be independent of spinal cord injury. Indeed, contrary to our second hypothesis, we found few outcomes that significantly worsened with duration or severity of injury. Moreover, nearly all functional systolic and diastolic outcomes reported herein are well within the range of normal values according to the ASE: ejection fraction, strain, and passive diastolic filling, for example, were all within the healthy range and did not significantly change with duration or severity of injury. This chronic spinal cord injury cohort is the largest to our knowledge study myocardial changes after spinal cord injury, and the largest sample of females with spinal cord injury. This indicates decreased cardiac outcomes in individuals with spinal cord injury may be a functional adaptation: spinal cord injury in isolation may not drastically accelerate myocardial remodeling that leads to systolic and diastolic dysfunction as previous reports suggest.

### Left-sided measurements

Measurement of the left ventricle during diastole and systole reflects not only preload and afterload within the left ventricle, but also the thickness of the myocardium and its ability to generate force and pump blood into the high-resistance systemic circulation. Cardiovascular disease can manifest as progressive thinning of the myocardium, which would lead to decreasing myocardial dimensions, decreased left ventricle mass, and ultimately lead to systolic and diastolic dysfunction whereby the heart cannot adequately pump blood to maintain homeostasis [46, 47]. This is reflected in progressively increasing left atrial dimension, due to accumulation of blood volume as the left ventricle weakens [48].

Spaceflight–frequently used in cardiovascular spinal cord injury research as a model of severe spinal cord injury due to skeletal muscle unloading–for as little as four hours can lead to rapid decreases in left ventricular volumes, while unloading of skeletal muscle for just 30 days can lead to significant decreases in left ventricular mass, fractional shortening, and contraction velocity that increase risk of orthostatic intolerance [10–14]. Individuals with chronic spinal cord injury have been reported to experience decreased left ventricle mass, diastolic internal dimension, septal and posterior wall thickness when compared with able-bodied controls [1–4, 39]. However, these significant decreases were a means-comparison between age-matched, able-bodied individuals that were not accompanied by decreases in systolic or diastolic function, and the relation to level, duration, or severity of injury is unknown. We found similar decreases in left ventricular mass and dimensions in female individuals compared with males, and individuals with thoracic spinal cord injury compared with cervical; however, cardiac dimensions were still within the range of ASE-determined healthy values and there were no meaningful significant changes with duration or severity of injury. The smaller chamber was not accompanied by abnormal left atrial volumes or dimensions, indicating there were no resulting deleterious decreases in myocardial force. Likewise, even though Z-scores indicate relative wall thickness and posterior wall dimension are skewed when compared with normative data, these increased outcomes are not associated with increased left ventricular mass, increased interventricular septum dimension, or systolic blood pressure. Concentric remodeling of the heart, associated with increased myocardial dimensions and increased relative wall thickness, occurs as the myocardium thickens in response to prolonged hypertension. It’s therefore unlikely these changes result from maladaptive myocardial remodeling. More research is warranted to understand these myocardial adaptations after spinal cord injury and their implications for cardiovascular health.

Given how rapidly the myocardium adapts to unloading, and that we found no significant relationship to duration and severity of injury, our data suggest the decreases in left ventricular mass and dimension are not the result of progressive decreases in function but rather a functional adaptation to the decreased preload and afterload that accompany spinal cord injury. End diastolic volume was significantly less in women compared with men, and when comparing thoracic spinal cord injury with cervical we found lower end diastolic volume in thoracic spinal cord injury (though it was not significant). This is supported by research performed by Sharif, et. al., who loaded the left ventricle of individuals with spinal cord injury with boluses of saline and found the myocardium was able to stretch without significant increases in filling pressure and was thus not fibrotic. Therefore, the smaller LV dimensions and decreased filling ratios reported by other researchers may result from functional adaptation which could explain why overall diastolic function was preserved.

### Systolic function

Systolic function outcomes reflect the ability of the left ventricle to generate sufficient force to pump blood into the systemic circulation. Systolic dysfunction can initially present as progressively decreasing ejection fraction, VTI, cardiac output, and stroke volume, and increasing end systolic volume [49] as the weakening myocardium struggles to pump blood. Subclinical myocardial systolic dysfunction can be detected by strain imaging (i.e., the percent decrease in myocardial size during systole relative to diastole), and in the non-injured population decreases in strain and torsion of the left ventricle often predict systolic dysfunction and can be seen before drastic decreases to cardiac output are evident [50, 51]. Because of this, decreases in strain, cardiac output, ejection fraction, and stroke volume are significant predictors for future cardiovascular events [52].

We found global circumferential and longitudinal strain were above the normal limits (i.e., more negative) and did not significantly change with duration, level, or severity of injury, indicating there were no significant decreases in myocardial contractility related to injury. In general, individuals with spinal cord injury demonstrate decreased cardiac output and stroke volume compared with non-injured persons, and some rat models of spinal cord injury demonstrate decreased contractility [32, 33, 53]. We did find significantly decreased cardiac output and stroke volumes in women compared with men, and stroke volume was below the ASE-determined range for healthy while Z-scores for indexed cardiac output indicate these values are skewed when compared with normative data. However, the decreased cardiac output and stroke volume was not significantly related to injury and was neither accompanied by progressive decreases in ejection fraction nor increases in end systolic volume. Decreased volumes without concurrent decreases to strain and ejection fraction are more likely to reflect changes in preload via the Frank-Starling mechanism. It is therefore likely the decrease in cardiac output and stroke volume, particularly as they are independent of injury, is an adaptation (e.g., reduced preload, decreased metabolic demand) and does not illustrate a progressive decline in systolic function.

### Diastolic function

Diastolic function reflects cardiac health during diastole, illustrating elasticity of the ventricles by measuring filling pressure and volume in the atria and ventricles. Diastolic dysfunction is marked by increased stiffening of the ventricles that compromise passive filling and relaxation, leading to progressive increases in left atrial pressure and volume that can drastically increase risk for heart failure [52]. Diastolic dysfunction can result from increased afterload due to age-related stiffening of blood vessels and hypertension [50, 51]: pressure within the left ventricles does not drop rapidly enough in early diastole, causing a load-dependent delay in cross-bridge inactivation and incomplete relaxation [54, 55]. This stiffening leads to decreased filling during early diastole relative to late diastole as the ventricles rely on atrial contraction to contribute blood to preload and increases the duration of isovolumic relaxation time. Decreased E-wave velocity relative to A-wave velocity, a decreased E/A ratio, decreased E-wave deceleration time illustrate progressive stiffening of the left ventricle, while increased isovolumic relaxation time and decreased e’ velocity illustrate progressive impairments in relaxation of the myocardium.

Others have reported individuals with spinal cord injury demonstrate decreased passive filling and E/A ratio compared to controls [1, 2]: Druissi and Matos-Souza both reported a shift in diastolic filling from early to late diastole, but outcomes were insufficient to be graded as diastolic dysfunction because the E/A ratio, isovolumic relaxation time, and mitral deceleration time were within healthy range. Our individuals with spinal cord injury had E-wave and A-wave velocities, isovolumic relaxation time, and myocardial relaxation velocities within the ASE-healthy range and did not worsen with duration, level, or severity of injury. Mitral valve deceleration time in male individuals with spinal cord injury was greater than ASE values, and the Z-scores indicate these values are skewed when compared with normative values. However, increased mitral valve deceleration time alone, i.e., without associated changes to filling velocities, E/A ratio, or isovolumic relaxation time, is not a clinically significant indicator of diastolic dysfunction. It is more likely related to heart rate: as heart rate decreases, such as with individuals with spinal cord injury, duration of diastole increases along with the duration of passive filling, which will increase mitral valve deceleration time.

In the non-injured population, diastolic dysfunction correlates significantly to afterload and worsens with age, and animal models have demonstrated afterload-dependent impairment to relaxation can occur immediately [54, 55]. Even though our diastolic function outcomes were clinically healthy, there was a significant increase in both septal and lateral E/e’ ratio with systolic blood pressure. Likewise, A-wave velocity and isovolumic relaxation time increased significantly as age increased. This illustrates the relationship between diastolic filling pressure, systolic blood pressure, and age is still evident in spinal cord injury, even when individuals are young and hypotensive and do not yet experience load-dependent delays in relaxation. In order to determine if these age-related changes are accelerated in spinal cord injury and how quickly they develop in a population that’s largely hypotensive, a larger study sample is needed.

It is important to note these measurements were obtained according to ASE guidelines in the left lateral decubitus position. Guidelines and ranges used to assess presence of systolic and diastolic dysfunction in order to determine risk of myocardial infarction or coronary heart failure are established in the supine position [45]. While we were unable to determine any meaningful relationship between sympathetic impairment and cardiac function in chronic spinal cord injury, it has been found in rodent models [32, 33]. It is possible deficits to cardiac function may be discovered in response to a stress mediated by the sympathetic nervous system (e.g., pharmacological, postural hypotension, etc.). Confounding this, however, is the fact that there are no ASE guidelines that define a healthy range outside this condition because it is established that many indicators of systolic and diastolic dysfunction (i.e., global strain, ejection fraction, e’ velocity, E/A ratio, etc.) are preload and afterload dependent [56–58]. Future research is warranted to compare the differences, if any, that emerge during with alterations to preload and afterload in chronic spinal cord injury with able-bodied individuals.

## Conclusions

Our data demonstrate that, despite significant differences in cardiac structure and function due to biological sex, there are no significant declines in function that can be attributed to duration, level, or severity of injury. Moreover, nearly all outcomes were within the ASE criteria for healthy. This is an important finding because, when comparing spinal cord injury individuals to non-injured individuals, many have found significant decreases to left ventricular mass, ejection fraction, cardiac output, E-wave filling velocity, and e’ relaxation velocity and they have been attributed to injury [1, 2, 4–7]. However, when comparing these values to the ASE guidelines, many are reporting significant cardiac abnormalities in spinal cord injury when the values are within normal range. This raises the question in spinal cord injury research, can a significant reduction in outcomes compared to a matched, non-injured sample be a clinically significant dysfunction if all the values are within normal range and do not get demonstrably worse as duration of injury increases?

This is not to undermine the large degree of cardiovascular dysfunction experienced by individuals with spinal cord injury: sympathetic impairment leads to blood pressure instability that drastically decreases quality of life by decreasing autonomy and independence and delaying application of therapeutic interventions [59–61]. However, it is possible the degree to which spinal cord injury leads to systolic and diastolic dysfunction may be overstated. This is a promising finding: decreases in cardiac systolic function experienced by individuals with spinal cord injury may be an adaptation rather than an irreversible loss in myocardial systolic function. Therefore, therapies that target cardiovascular regulation (i.e. mitigation of persistent hypotension, orthostatic hypotension, and/or autonomic dysreflexia) in this population are quite likely not only to be successful, but likely to increase self-reliance, autonomy, and quality of life, and lead to long-term decreases in cardiovascular morbidity and mortality.

### Limitations

Our data reflect resting cardiac structure and function obtained from individuals with spinal cord injury healthy enough to enroll in a clinical trial. To mitigate sampling bias, eligible individuals were contacted from a nationwide database of individuals with spinal cord injury. As such, our cohort includes a sample of individuals with spinal cord injury with varied demographics, injury characteristics, and care plans more representative of the entire population than if we recruited directly from an outpatient hospital or wellness facility. Despite this, information needed to account for activity-based therapy or exercise regimens, residual sympathetic and/or motor activity, plasma triglycerides, etc., was unavailable.

Individuals with chronic spinal cord injury also spend most of their day seated upright in their wheelchair. Obtaining cardiac ultrasound in the upright position (while accounting for limitations that will occur from suboptimal images) can lend insight into hemodynamics in chronic spinal cord injury and better target interventions to restore cardiovascular function.

Although the average age of our cohort was relatively young (36 ± 12 years), younger patients have less cardiovascular risk factors allowing more accurate comparisons for the potential effects of chronic spinal cord injury. There was also an unequal distribution of injury characteristics, and despite our generous recruitment of women, our sample still had a greater proportion of male, cervical, motor-complete injuries. However, this is more representative of the spinal cord injury population who are more likely to be males with cervical, complete injuries.

## Supporting information

S1 Data (XLSX)

S1 File (XLSX)

S1 Table Left side chamber size, geometry, and mass mean values and Z-scores from individuals with spinal cord injury and ASE normal values. (XLSX)

S2 Table Global systolic function and blood pressure mean values and Z-scores from individuals with spinal cord injury and ASE normal values. (XLSX)

S3 Table Diastolic function mean values and Z-scores from individuals with spinal cord injury and ASE normal values. (XLSX)
